# Supplementary material for: Diagnostic and Prognostic Potential of Exosomal Cytokines IL-6 and IL-10 in Polytrauma Patients
Source: Int J Mol Sci. 2023 Jul 23;24(14):11830. doi: 10.3390/ijms241411830 (PMC10380769; doi:10.3390/ijms241411830)
Supplement: Supplementary file 1 [file ijms-24-11830-s001.zip › ijms-2445986-supplementary.pdf]

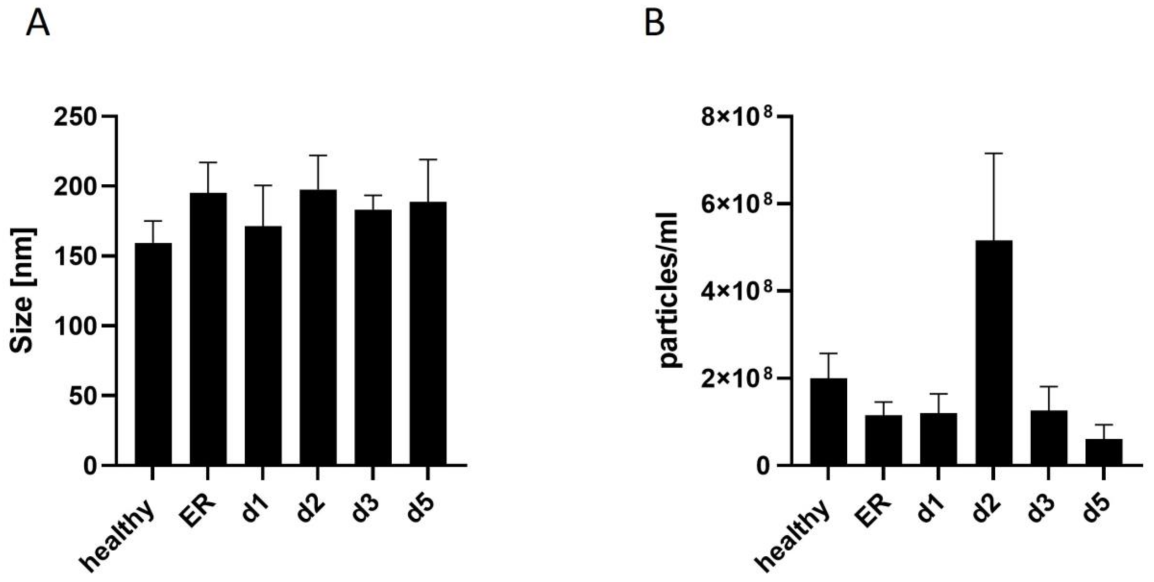

Supplemental Figure S1. Nano tracking analysis of exosomes: A) Mean size of exosomes measured via NTA in healthy and polytrauma patients at different time points; B) Particles numbers measured via NTA.
